# A Practical Workflow for Correcting Kit-Specific Effects in Whole-Exome Sequencing Data

**DOI:** 10.3390/mps9050125

**Published:** 2026-08-25

**Authors:** Laura Jarosz, Marcel Ochocki, Julia Merta, Lajos Pusztai, Michal Marczyk

**Affiliations:** 1 Department of Data Science and Engineering, Silesian University of Technology, 44-100 Gliwice, Poland; laura.jarosz@polsl.pl (L.J.); marcel.ochocki@polsl.pl (M.O.); julia.merta@polsl.pl (J.M.); 2 Yale Cancer Center, New Haven, CT 06511, USA; lajos.pusztai@yale.edu

**Keywords:** batch effect, whole-exome sequencing, exome capture kit, imputation, genetic variants

## Abstract

Large-scale, multi-center projects have become common in the era of rapid technological development, but protocol standardization remains challenging. In whole-exome sequencing (WES), various exome enrichment kits exhibit variable efficiency across genomic regions, leading to systematic, non-biological batch effects, much stronger than other technical factors. We propose a workflow to minimize the effect of WES capture inconsistencies in single-nucleotide variation (SNV) data. The pipeline consists of quality control, mapping to the genome, SNV calling, joint genotyping, and imputing genotypes using reference haplotypes. SNVs are then aggregated into gene-level features measuring the burden of deleterious variants. Finally, a gene-level imputation is performed using a customized algorithm. Namely, if the detection rate of a gene is low in samples enriched with a given capture kit but high in samples enriched with other kits, missing values in the former group are imputed, as such differences are unlikely to reflect true biology. As a benchmark, we conducted a study on over a thousand breast cancer cases across 11 cohorts, using eight exome capture kits. We demonstrated that the proposed pipeline leads to a considerable decrease in the batch effect signal, potentially increasing the likelihood of finding true biological signals.

## 1. Introduction

Technological advances in recent decades have enabled large-scale sequencing projects, allowing researchers to analyze numerous, diverse cohorts [1]. The 1000 Genomes Project (1kGP) is a pioneering population-level initiative that provided one of the first global references for human genetic variation [2]. A more cancer-focused example is The Cancer Genome Atlas (TCGA), which characterized the genomic landscape of 33 cancer types by collecting over 20,000 samples [3]. The most recent project is the UK Biobank, which includes over 500,000 participants, making it one of the largest genetic data resources [4]. Despite the invaluable benefits of expanding analytical capabilities, these projects also have some drawbacks. When designing a study at such a large scale, incorporating multiple sites is often necessary to ensure feasibility. For example, there are around 17 million containers with blood, urine, and saliva specimens held in the UK Biobank laboratories, highlighting the logistical complexity and infrastructure required to succeed with such a venture. As the number of collaborating sites increases, protocol standardization becomes more challenging [5]. The procedure of carrying out the experiments and processing hundreds of samples in different environments introduces technical variability, known as batch effects. This non-biological variation may considerably confound the results and lead to incorrect conclusions [6].

Whole-exome sequencing (WES) enables access to all protein coding sequences of the human genome, significantly reducing the costs and computational effort compared to whole-genome sequencing (WGS) [7]. In WES, technical variability can arise at multiple stages of the study. For example, a specimen preservation method involving fixation in formalin and embedding in paraffin (FFPE) is known to induce cytosine deamination, leading to C > T transition artifacts [8]. During library preparation, differences in PCR can cause amplification biases, such as sequencing duplicates or inaccurate representation of the original sequence—especially in GC- and AT-rich regions [5,9]. Different tools used in the bioinformatic pipeline may lead to divergent results in SNV detection [10]. Finally, the use of different exome capture kits results in systematic coverage variability, with inefficiencies in certain target regions and an increased false negative rate [11]. Recently, we compared multiple technical and clinical characteristics of the patient samples and found that the highest variance was due to the capture efficiency differences between the various versions of the kits, which led to systematic gene-level detection biases in WES data [12]. These biases manifest as near-binary gene detection patterns, where variants in a given gene are discovered in almost all the samples enriched with one capture kit but none of those processed in another.

There are numerous methods for batch effect correction in transcriptomics. ComBat reduces the effects of known batches using the empirical Bayesian framework [13]. Limma facilitates batch effect removal by applying linear modeling techniques [14]. The Harmony method, designed for single-cell RNA sequencing, adjusts for the batches while preserving biological signals [15]. However, to date, we are not aware of any comprehensive, dedicated framework to overcome these issues in SNV data, since their nature is somewhat different from transcriptomic data. Nevertheless, there are some methods that can be used to reduce some specific technical artifacts. Differential depth of coverage can be normalized through downsampling the overrepresented reads. GC-bias can be corrected with a dedicated model [16]. Multiple tools have been developed for dealing with artifacts in FFPE samples [17,18,19,20]. However, none of the described methods can be applied to WES SNV data in terms of addressing the variability arising from differences in exome capture. To fill this gap, we propose a workflow aimed at minimizing batch effects primarily driven by differential enrichment efficiency in WES SNV data.

Our pipeline starts conventionally, with quality control of raw sequencing reads, trimming, and mapping to the reference genome. The next step consists of calling the SNVs and jointly genotyping the samples with a restriction to the custom regions of the genome. SNVs are then post-processed and prepared for the genotype imputation with the reference haplotype method. Subsequent aggregation into gene-level average allele fraction weighted by the CADD Phred-like score allows measuring the burden of deleterious SNVs within the genes. Finally, a gene-level feature imputation is performed, applying our custom algorithm. The core rule states that if the detection rate of the gene is extremely low in samples processed with the same exome capture kit but substantially higher in samples enriched with other kits, the missing values in the former group are imputed, as such differences are unlikely to reflect true biology. For benchmarking purposes, we conducted a study on over a thousand breast cancer cases across 11 cohorts enriched using eight exome capture kits.

## 2. Materials and Methods

### 2.1. Pipeline Overview

**Figure 1:**
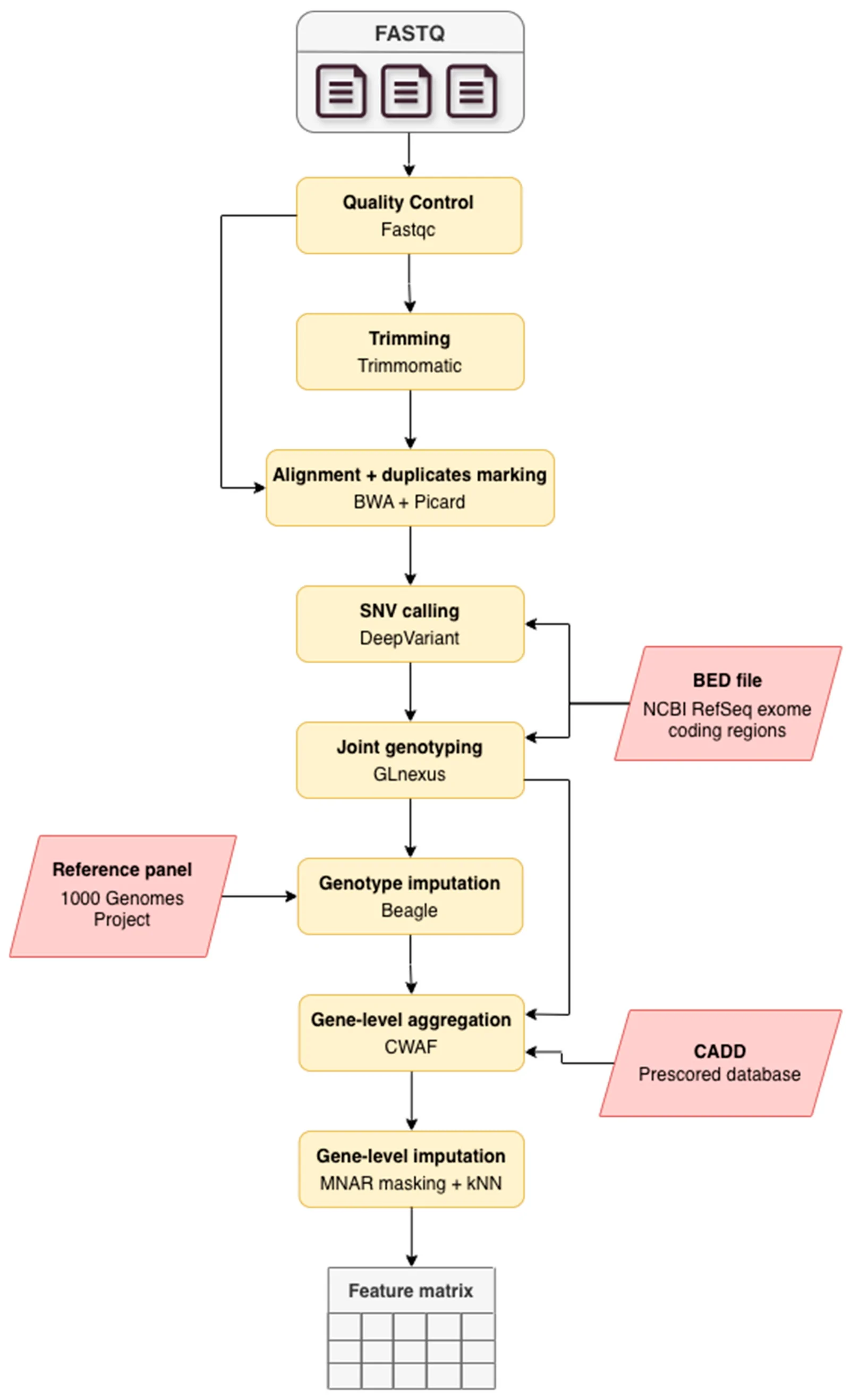
Overview of the proposed pipeline. CWAF, CADD-weighted average allele fraction; MNAR, missing not at random; SNV, single nucleotide variation. MNAR masking identifies gene-cluster combinations with likely technical under-detection, which are subsequently imputed using kNN.

The proposed method (Figure 1) is an end-to-end framework that integrates all processing steps from raw sequencing reads to a gene-level feature matrix, explicitly reducing batch effects driven by differences in exome capture efficiency. The pipeline is initiated by a quality check (QC) of FASTQ files with a focus on sequence/base quality scores and adapter content. Depending on the QC results, reads are either trimmed first or directly aligned to the reference genome, followed by duplicate marking. Subsequently, SNVs are detected with DeepVariant, which generates both VCF and GVCF files for each sample independently [21]. GLnexus is utilized to merge and genotype GVCFs into a single cohort-level VCF [22]. Both of these steps are restricted to regions specified in a BED file built on NCBI RefSeq exonic coordinates [23]. Genotype imputation is carried out using Beagle with a reference panel built from phased VCF files from the 1kGP [2,24,25]. The feature calculation step utilizes both imputed and non-imputed files, as well as the CADD prescored database, aggregating the SNVs into a CADD-weighted average allele fraction (CWAF) [26]. Subsequent feature imputation is performed on the gene-level matrix (samples × genes), with a mask defining missing-not-at-random (MNAR) entries, which are selectively imputed. The implementation supports parallel execution across samples or chromosomes, depending on the current step. The code of the workflow is publicly available here: https://github.com/ZAEDPolSl/WESworkflow (accessed on 20 August 2026).

### 2.2. Quality Control and Mapping of Reads

The quality check of the raw sequencing reads is carried out with fastQC (v0.12.1) and MultiQC (v1.34) is used for reporting [27]. Low-quality reads are trimmed with Trimmomatic (v0.39; SLIDINGWINDOW:4:20), removing the 3′ end when the average Phred quality within a 4-base window drops below 20. Reads shorter than 45 bp after trimming are discarded, and if detected, adapters are removed (ILLUMINACLIP) [28]. The Burrows–Wheeler Aligner (BWA; v0.7.17-r1188) is used for mapping to the reference GRCh38.p14 genome (Ensembl primary assembly release 109) with *−T 0* flag to increase sensitivity [28,29]. The resulting SAM files are converted to BAM format and coordinate-sorted with Samtools (v1.19.2) [30]. Sequencing duplicate marking is performed with Picard MarkDuplicates (v3.3.0) to ensure redundant reads are ignored in downstream steps [31]. Additionally, for data available only as GRCh37-aligned BAM files, coordinates are lifted over to GRCh38 using CrossMap (v0.7.0) with an Ensembl chain file (25-07-2014) [32].

### 2.3. SNV Calling and Joint Genotyping

Exonic regions were derived from the UCSC hg38 refGene database (release 17-08-2020). Coordinates with ±5 bp flanking were extracted from transcript records and merged to obtain a non-redundant set of genomic intervals, excluding non-canonical contigs and patch sequences. The resulting BED file is used to restrict SNV calling and joint genotyping to target regions, thereby reducing the computational effort and improving scalability.

For the SNV detection step, DeepVariant (v1.8.0, model_type = WES) is applied [21]. GVCF output is enabled by default, and the resulting per-sample files are subsequently merged and jointly genotyped with GLnexus (v1.4.1-0-g68e25e5; DeepVariantWES config), yielding a cohort-level, multi-sample VCF.

### 2.4. Genotype Imputation

During the pre-imputation processing, multiallelic sites in the multi-sample VCF are split, SNVs extracted, left-aligned, and records with at least one sample having alternative allele depth (AD) > 5 are retained. The multi-sample VCF is partitioned by chromosome, with all subsequent steps up to feature imputation executed in parallel, only for computational purposes. Genotype imputation is carried out with Beagle (v5.5), which has been shown to considerably reduce the computational cost without sacrificing the precision [24]. To ensure maximal concordance with the imputation reference panel, query genotypes are conformed by allele matching and strand alignment, with unresolved records being excluded. Subsequently, haplotypes are phased across the cohort and used for genotype estimation and imputation of missing SNVs [25].

The reference panel was constructed from 1000 Genomes Project VCF files (30x on GRCh38), for each chromosome separately [33]. Samples (n = 3202) were restricted to individuals of European ancestry (n = 632) to match the benchmark cohort and maximize imputation accuracy, as haplotype structure varies considerably across populations [34]. The instruction that describes a complete procedure starting from downloading the phased VCF files, followed by ancestry-based sample selection, chromosome name conversion, SNV filtering, indexing, and conversion to BREF3 format required by Beagle is provided in our GitHub repository. A reference panel can also be constructed for other ancestral groups.

### 2.5. SNVs Post-Processing and Filtering

As a result of genotype imputation, all SNVs present in the reference panel are represented in the output VCF, including those previously absent in the query cohort. Consequently, the imputed VCF contains additional records, while those unresolved during genotype conforming are absent from the final dataset. Therefore, directly observed SNVs present in the non-imputed VCF are annotated using ANNOVAR (version 02-03-2025) with multiple annotation sources (Table A1) and the CADD prescored database (v1.7) [35,36]. The resulting VCF files are filtered to retain SNVs annotated as *exonic*, *splicing*, *exonic;splicing*, or *ncRNA_exonic;splicing*. This procedure yielded a final set of SNVs selected for downstream analyses. The imputed VCF is subsequently restricted to this SNV set, resulting in a substantial reduction in file size and computational burden in downstream processing.

### 2.6. Gene-Level Aggregation

Missing CADD Phred-like scores are imputed using k-nearest neighbors (kNN; *k* = 5) implemented in *scikit-learn* (v1.7.2) Python (v3.12.12) library [37]. The algorithm is run using additional SNV-level features, namely, global allele frequency, SNV type, MetaSVM prediction, and ClinVar significance annotations, to provide potentially better imputation.

For each sample, SNVs are aggregated per gene with the information derived from both the original and imputed VCF files. Allele fractions (*AF*) are calculated primarily as the ratio of alternative allele read counts (*ALT*) to the total allelic depth (sum of reference (*REF*) and alternative allele counts) at a given position (AF=ALT/(REF+ALT)). In cases where *AF* could not be determined, estimated allele dosage (DS) from the imputed VCF is used (AF = DS/2). If this measure is also unavailable, *AF* is set to zero. When germline SNVs are analyzed, SNVs having *AF* > 0.2 are retained in further analysis.

For each gene and sample, a CADD-weighted average allele frequency (CWAF) was computed as the weighted average of SNV-level AF values with CADD Phred-like scores serving as weights (1):

(1) $$CWAF_{g,p}=\frac{\Sigma_{i\in g}AF_{i,p}\;\cdot \;CADD_{i}}{\Sigma_{i\in g}CADD_{i}}$$

where *g*,*p* denote gene and patient, and *i* indexes SNVs within gene *g*. The resulting gene-level feature matrix (samples × genes) was used for downstream analyses.

### 2.7. Gene-Level Feature Imputation

Gene-level imputation is performed in an R (v4.5.1) environment. Subsequently, samples are clustered using *PARC* (v0.40; *knn = 30*, *jac_std_global = 0.15*, *resolution_parameter = 1*, *random_seed = 42*) implemented in Python [38].

**Figure 2:**
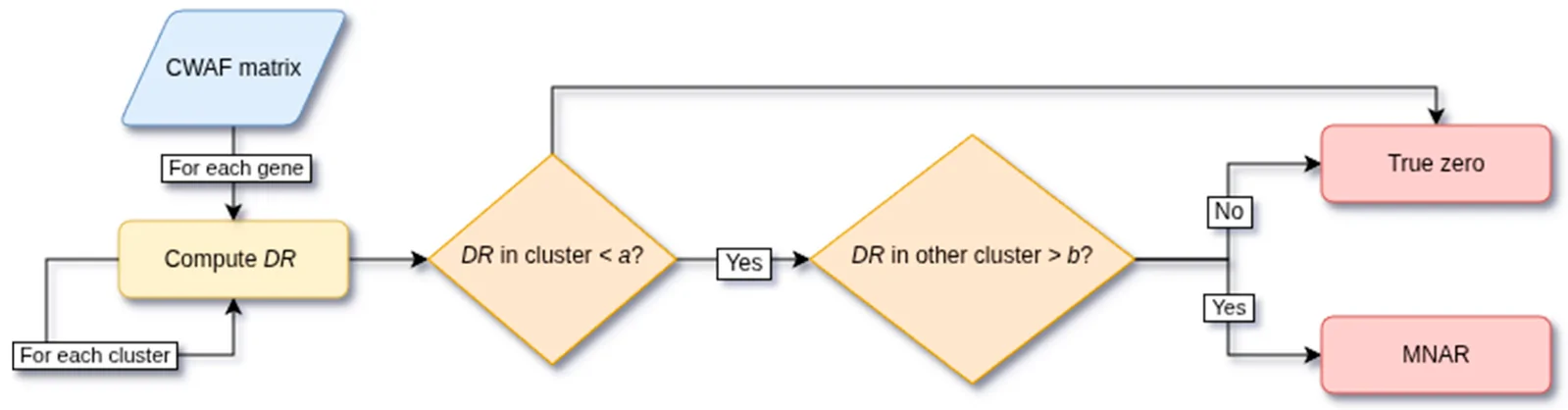
MNAR masking scheme. CWAF—CADD-weighted average allele frequency. DR—detection rate.

A missing-not-at-random (MNAR) mask is defined using gene-level detection rates (DRs) across clusters. The DR is computed as the proportion of samples with non-zero CWAF values per gene and cluster. Gene-cluster pairs with a low DR (DR< a) in a given cluster and a high DR (DR > b) in at least one other cluster with at least 30 members are flagged as under-detected. These flags are propagated to the sample level, and only missing CWAF entries corresponding to flagged gene-cluster groups are marked as MNAR (Figure 2). Thresholds a and *b* are estimated by modeling the DR across all the clusters as a Gaussian mixture using *dpGMM* (v0.1.9) [39].

Gene-level imputation is carried out using a masked cosine similarity-based kNN approach. For each sample and MNAR entry, samples from the same cluster are excluded from candidates from which to impute to avoid within-cluster bias. Cosine similarity is computed using non-MNAR genes shared between candidates and the sample to impute, thereby preventing similarity driven by shared missingness. The minimum overlap (*min_overlap = 50*) of common features is required. The nearest neighbors (*k = 5*) are used to impute a similarity-weighted average. The procedure is parallelized across samples using the *future* package [40].

### 2.8. Benchmark Study

#### 2.8.1. Samples and Data

A total of 1077 samples from 11 datasets enriched with 8 exome capture kits were analyzed (Table 1). WES data were obtained from germline breast cancer samples of Caucasian ancestry.

#### 2.8.2. Benchmark Analysis

The workflow described in Section 2.2, Section 2.3, Section 2.4, Section 2.5, Section 2.6, Section 2.7 and Section 2.8 was applied with default settings. Gene-level aggregation (2.7) was additionally performed, omitting the information from the imputed VCF, thereby enabling us to benchmark genotype imputation performance.

To conduct the visual assessment of the underlying CWAF structure, uniform manifold approximation projection (UMAP) was used with the R package *uwot* (v0.2.3; *n_neighbors = 15*, *min_dist = 0.5*, *metric = cosine*, *n_epochs = 1500*, *nn_method = nndescent*). Additionally, two batch effect metrics were computed to evaluate the performance of the correction methods, with the same set of parameters in all cases and the dataset serving as batch label. Local Inverse Simpson’s Index (LISI) obtained with lisi package (v1.0; *perplexity = 15*), quantifies batch mixing, with higher values indicating improved diversity of batches among the neighbors for a given data point [15]. The k-nearest neighbors batch effect test (kBET) performs Pearson’s $\chi^{2}$ test to compare the distribution of batches in the local neighborhood to their global distribution, rejecting the null hypothesis when significant dissimilarities are observed. The *kBET* (v0.99.6; *k0 = 15*, *heuristic = FALSE*, *do.pca = FALSE*) library was used for statistical testing, with the *RANN* package (v2.6.2; *k = k0 + 1*) utilized to compute the neighborhood matrix [41,42]. As reported in this study, values reflect the average LISI score or kBET null hypothesis rejection rate across all samples in the analyzed dataset.

Genes with a differentiated CWAF across clusters were evaluated using the Kruskal–Wallis test, and a false discovery rate (FDR) adjustment was applied to the resulting *p*-values. Top 10% significant genes identified for the data before the genotype and CWAF imputation were selected for subsequent analysis of DR.

Preservation of biological signal following gene-level imputation was assessed using *limma* R package (v. 3.23) [14]. Patients were assigned to two groups reflecting the average age of menopausal transition (<50 vs. ≥50 years). CWAF values were modeled as a function of age group while adjusting for technical cluster. The resulting *T*-statistics for the age group effect were compared before and after gene-level imputation using Pearson’s correlation. Additionally, the absolute change in *T* was evaluated in relation to DR across clusters (maximum DR–minimum DR).

### 2.9. GitHub Repository

The developed workflow has been implemented into a publicly available repository stored on GitHub: https://github.com/ZAEDPolSl/WESworkflow. The main README file serves as a clear entry point to the repository. It starts with a brief description of configuration and setup, provides a general overview of the workflow including all the performed steps and scripts, describes the repository structure and directs users to a detailed installation guide, external resource documentation, reference panel construction instructions, and a lightweight example run guide. The repository includes a dedicated Conda environment file including tools, R, and Python dependencies. For four R packages that cannot be resolved by Conda, there is an additional R installation script. A lightweight installation check script controls whether all necessary dependencies have been correctly installed and are available. To verify the installation of the repository, there are exemplary data from four small FASTQ files reconstructed from selected regions of public SEQC2 WES BAM files (the example dataset is intentionally reduced and is intended only as a technical execution test). To test the gene-level part of the pipeline, which requires a larger cohort structure, scripts to generate a separate artificial gene-level dataset are provided. All settings and paths are controlled using a YAML-based configuration system. In R scripts, all the adjustable parameters were extracted from the internal code and moved to the clearly specified section at the beginning of each script. Finally, there is a list of all required external resources together with download links, expected genome builds, versions, corresponding configuration key, and eventual required index files.

## 3. Results

### 3.1. SNV-Level Processing

For eight datasets, we were able to acquire raw FASTQ files, while for three cohorts (TCGA, EGAD00001002747, and EGAD00001003137), only BAM files were available. A total of 300 FASTQ file pairs were processed during QC of raw sequencing reads, of which 164 did not pass the control due to poor read-end quality. Low-quality reads were removed (BEAUTY, PRJNA516884) and adapter trimming was performed (EGAD50000000770) for these samples. Reads stored in BAM files were mapped to the GRCh37 reference genome; thus, for 775 files, we lifted over the genomic coordinates to the GRCh38 version. BAM quality metrics such as target coverage and duplicate rate were reported in Table S1.

In total, 1077 GVCF files were jointly genotyped, resulting in a single multi-sample VCF containing 483,851 records and 501,467 SNVs. During genotype imputation preprocessing, 10,816 multiallelic sites were split, and 13,139 SNVs were realigned to the reference, thereby obtaining a standardized representation of detected germline SNVs. After SNV extraction and allelic depth filtering, 440,344 SNVs were retained, corresponding to an 87.81% reduction in SNVs compared to the initial dataset. During genotype conformation to the reference panel, SNVs were removed from the query VCF, 69,966 due to reference mismatch and 3095 due to undetermined strand orientation. Genotype imputation recovered approximately 2% (n = 228,957) of missing genotypes across the analyzed cohort. Across 111 Beagle windows, the mean estimated genotype error rate was 0.31% ± 0.08% (mean ± SD). Among retained SNVs, the mean estimated dosage *R*^2^ *(DR2)* was 0.952, and 95.21% of SNVs had *DR2* ≥ 0.8.

### 3.2. Gene-Level Processing

#### 3.2.1. SNV Aggregation and Genotype Imputation Performance

**Figure 3:**
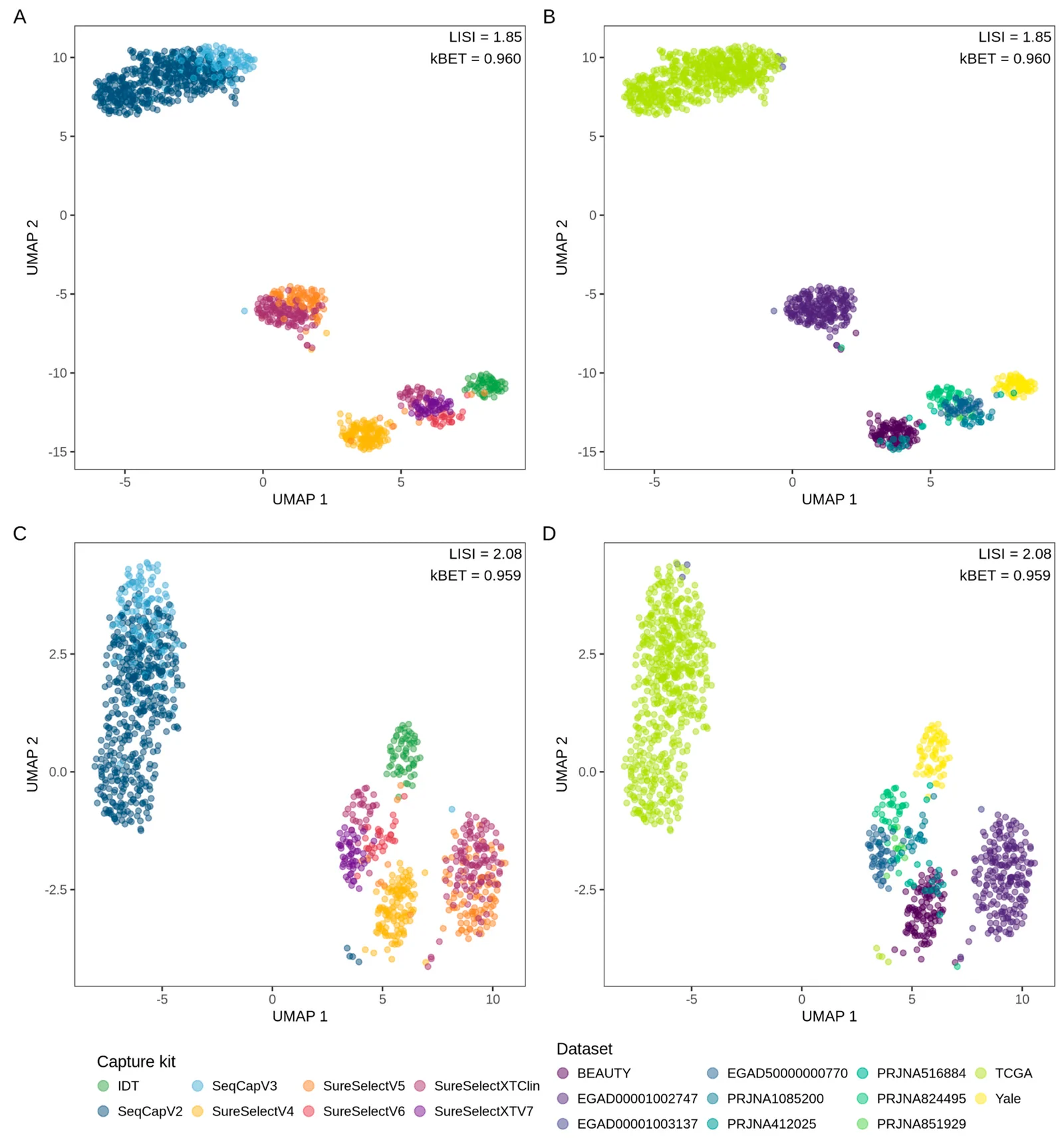
UMAP embeddings before (**A**,**B**) and after (**C**,**D**) genotype imputation, colored by: (**A**,**C**) exome enrichment kit; (**B**,**D**) dataset. Batch effects decreased as indicated by LISI improvement and kBet decrease.

Following the gene-level aggregation, a CWAF matrix of 1077 rows representing samples and 18,346 columns corresponding to genes with at least 1 SNV was obtained for both non- and genotype-imputed data. UMAP visualization revealed clearly separated groups, with TCGA samples enriched using SeqCap EZ capture kits forming the most distinct community and SureSelect/IDT samples being more similar (Figure 3A,B). Following genotype imputation, population structure became more diffuse, as reflected by an increase in LISI from 1.85 to 2.08, indicating reduced inter-group differences (Figure 3C,D).

#### 3.2.2. Sample Clustering

Several capture kits showed noticeably similar CWAF profiles, forming overlapping communities in the UMAP space, such as SureSelectV6, SureSelectXTV7, and partially SureSelectXTClin or SeqCapEZV2 and SeqCapEZV3. An interesting observation was made for SureSelectXTClin, where samples from the PRJNA824495 dataset exhibited evidently different locations than EGAD00001002747 samples generated with the same capture kit, reportedly (Figure 3C,D). Furthermore, most datasets were reportedly used exclusively for one exome enrichment kit, but individual samples were observed for groups with different kits than specified. We therefore hypothesized that these samples may correspond to externally processed cases included in the cohorts, or were possibly misannotated. Following these findings, samples were clustered to ensure that gene-level imputation was performed within the technically homogeneous groups sharing similar CWAF profiles rather than based on the provided kit annotations. Clustering parameters for genotype-imputed data were selected after analysis of nine *knn* and *resolution* combinations. Each time, the clustering results were projected onto the same UMAP embedding, choosing the configuration preserving major technical groups while avoiding excessive grouping. Several combinations yielded the same number of clusters preserving the major groups visible in the UMAP space while differing only in the assignment of a few individual samples, indicating stable major cluster structure (Figure S1). Finally, six clusters of samples (cluster nos. 0–5) sharing similar CWAF profiles were identified, comprising 374, 207, 197, 118, 115, and 66 members, respectively (Table 2).

#### 3.2.3. MNAR Masking and Gene-Level Imputation

**Figure 4:**
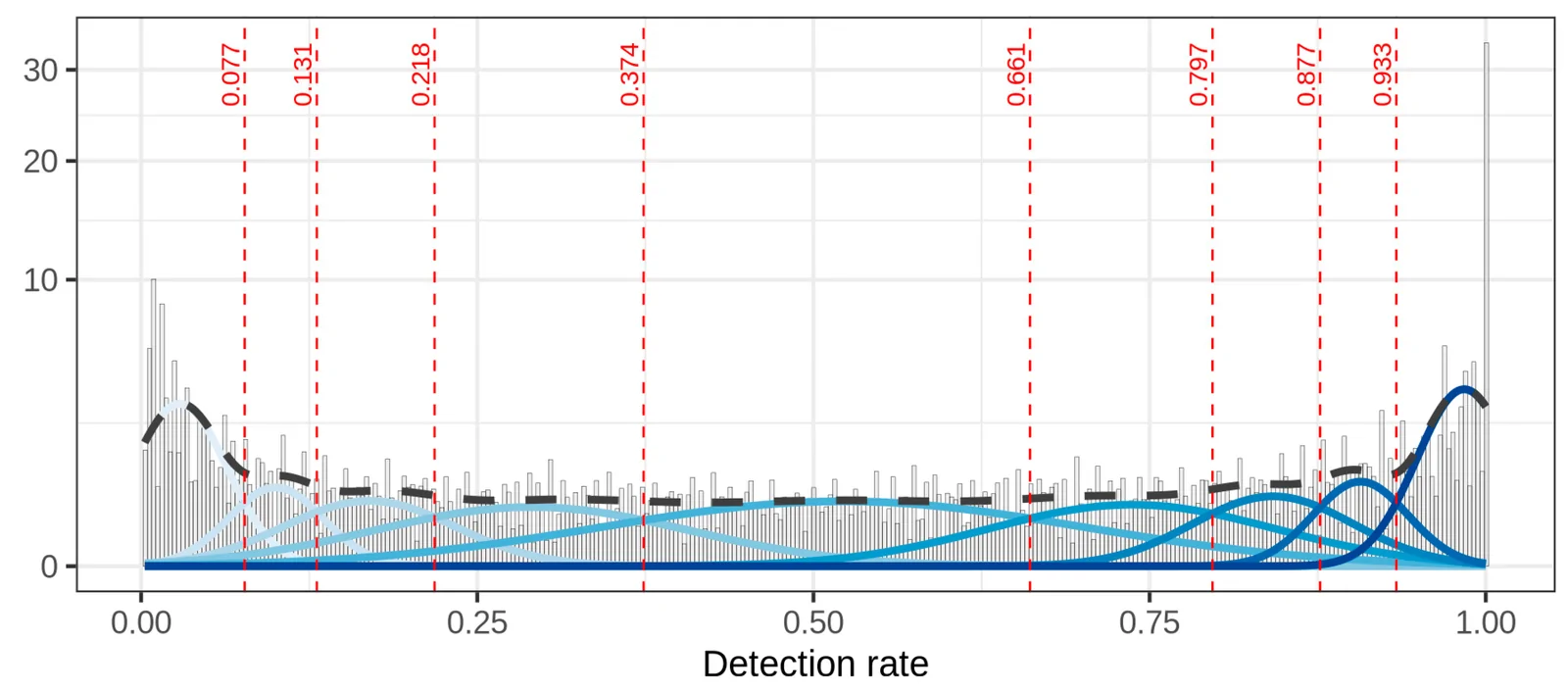
Gaussian mixture modeling of global gene DR. Blue curves represent individual model components, with the black dashed curve reflecting the full model. Estimated candidate thresholds for CWAF imputation were marked with a red dashed line.

For each gene, DR values across all groups were jointly modeled using a Gaussian mixture model, yielding eight thresholds at the intersections of adjacent components (Figure 4). To find optimal cutoffs for gene-level imputation, the grid search method was applied with $a\in \left(0.077,\;0.131,\;0.218\right)$ and $b\in \left(0.797,\;0.877,\;0.933\right)$.

**Figure 5:**
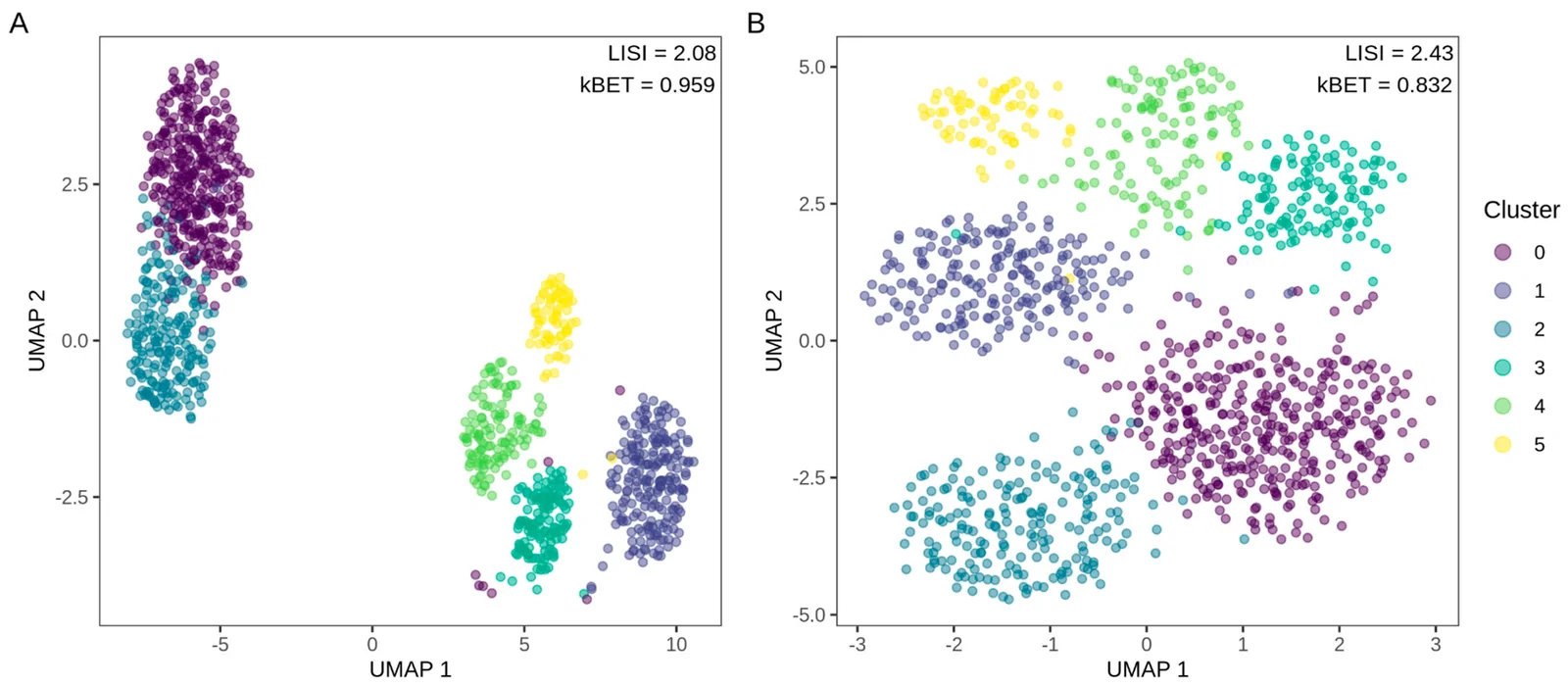
UMAP space colored by cluster assignments derived from CWAF values, before (**A**) and after (**B**) gene-level imputation. LISI increase (2.08 → 2.43) and kBET decrease (0.959 → 0.832) indicate successful batch effect reduction.

Sensitivity analysis was performed for all nine combinations of candidate thresholds. For each combination, batch effect metrics and the fraction of imputed entries were quantified. Increasing a or decreasing b relaxed the MNAR criterion, increasing the number of imputed values and generally improving LISI and kBET. As the fraction of imputed entries remained marginal across all combinations, ranging from 0.21% to 0.47%, the highest candidate value of a (*a* = 0.218) and the lowest of b (*b* = 0.797) were selected (Table S2). This yielded a 0.35 increase in LISI (2.08→2.43) and 0.127 decrease in kBET (0.959→0.832) (Figure 5). Imputed CWAF values accounted for 0.43% (n = 85,342) of all gene-level feature matrix entries. In the UMAP space, a considerably less pronounced separation between the clusters was observed, with samples forming a more integrated embedding (Figure 5B). Compared to raw data without the proposed imputations, the workflow resulted in an increase of 0.58 (31.35%) in LISI and a 0.128 (13.33%) decrease in the kBET rejection rate.

#### 3.2.4. Gene Detection

Given the previously reported nearly binary detection patterns across genes distinguishing exome capture kits [12], a comparison analysis was performed on the benchmark cohort. The evaluation was conducted at each processing stage: prior to imputation, following genotype imputation, and after gene-level imputation. CWAF differences between the clusters were assessed using statistical testing. Initially, 2875 genes showed significant differences, decreasing to 2705 after genotype imputation, and 2680 after CWAF imputation, yielding a total decrease of 195. The top 10% (n = 286) of initially identified significant genes were selected for the downstream DR analysis. For example, *FAM104B* was detected in >99% samples in cluster 1, 0.5% samples in cluster 2, and none of the samples in the remaining clusters. By contrast, genes like *KCNJ18*, *GPR179*, or *MRPL45* were detected in 96% to 100% of samples in clusters 3, 4 and 5, but had a DR = 0% in clusters 1 and 2. Four groups of detection patterns were found using k-means clustering. All top 10% significant genes are listed in Table S3.

**Figure 6:**
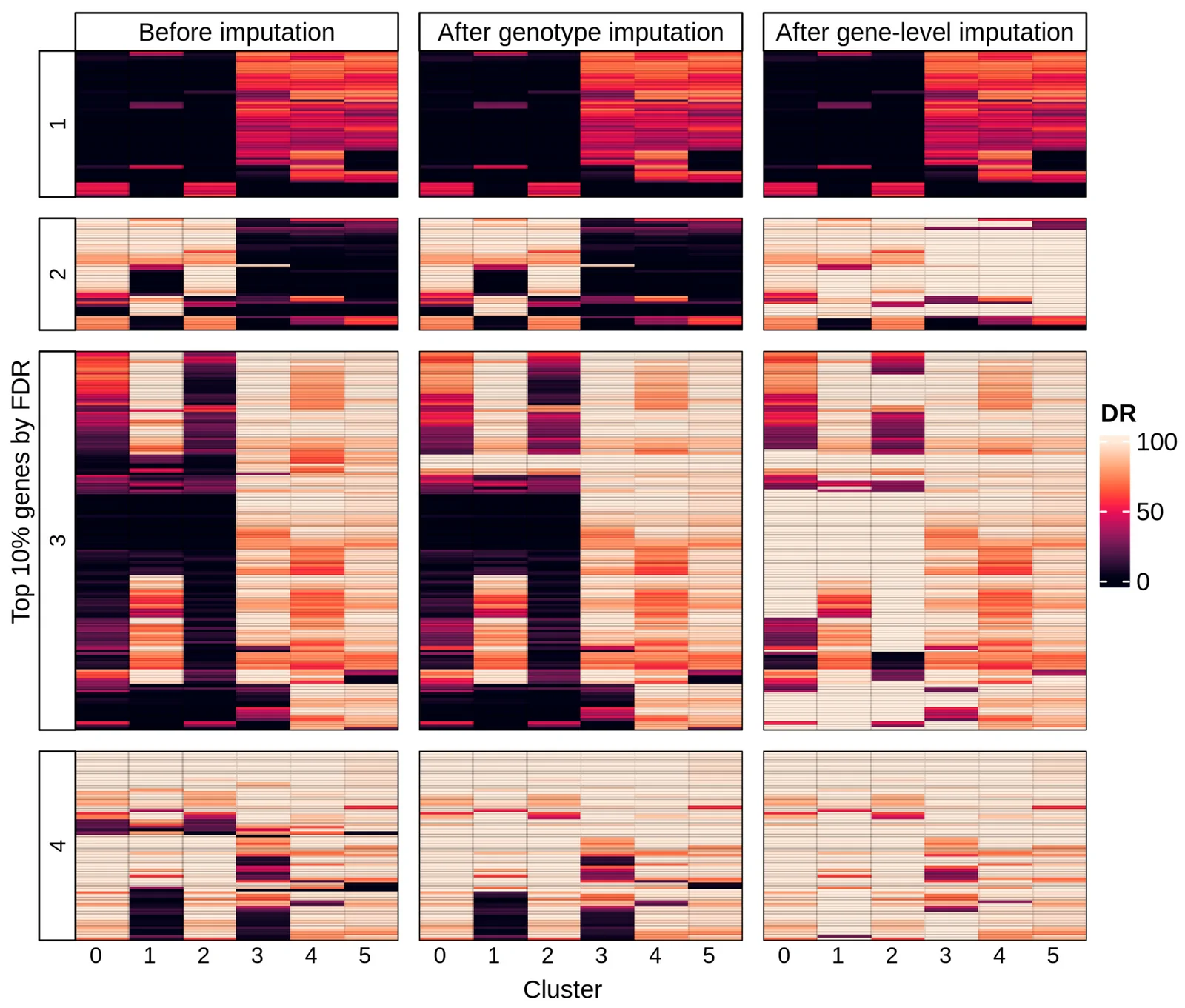
Comparison heatmap of gene detection rate (DR) across the sample clusters at each processing stage. In total, 10% of the most significant CWAF differentiating genes before the genotype and gene-level imputation were selected and clustered into 4 groups corresponding to heatmap rows.

We observed cluster-specific detection patterns, with the genes being detected in all the samples within the given cluster, but absent in others (Figure 6). Genotype imputation resulted in a modest DR improvement at the level of individual genes (Figure 6, rows 3–4). Gene-level imputation resulted in a noticeable global increase in gene detectability under the binary detection scenario (Figure 6, rows 2–3); however, as expected, it had no effect when DR differences between clusters were not sufficiently large (Figure 6, row 1).

To evaluate whether the gene-level imputation preserved the biological signal, age-associated effects were compared before and after the imputation. For EGA datasets, age metadata were not available; thus, 260 samples were not included in this analysis. Consequently, cluster 2 was also excluded from this step, as only one sample was left within this technical group. Patients were classified into two groups reflecting the pre- and post-menopausal age. T-statistics obtained from limma models before and after gene-level imputation were highly concordant, as reflected by Pearson’s *r* = 0.999. The absolute change in T-statistic was generally small (median $\left|\Delta T\right|=8.95\;\times 10^{-7}$; 95th percentile $=1.49\;\times 10^{-5}$). Only a small fraction of genes exhibited larger changes, with the 99th percentile reaching 0.083. Those genes were mostly characterized by greater inter-cluster differences in detection rate (Figure S2), suggesting successful retrieval of important biological signal due to imputation.

## 4. Discussion

Our work emphasizes the need for addressing the technical variability arising from the use of different exome enrichment kits. We reported that at the gene level, these biases manifest as near-binary gene detection patterns, where SNVs in a given gene are discovered in almost all samples enriched with one capture kit but none of those processed in another, highlighting the gene-level imputation importance [12]. There are dozens of exome capture kits available on the market, with each manufacturer offering multiple versions. The largest producers include Roche (formerly NimbleGen), Agilent, and Illumina, with more recent platforms provided by Twist Bioscience and Integrated DNA Technologies (IDT). In another study investigating four solution-based exome capture kits, Sulonen et al. report that regions captured with NimbleGen kits aligned more accurately with their targets, compared to Agilent methods. However, none of the considered kits captured all the Consensus Coding Sequence (CCDS) annotated exons [43]. Shigemizu et al. demonstrated that the Agilent XT method outperforms in target enrichment efficiency and sensitivity, while Illumina and NimbleGen excel in clinical application purposes [44]. In the most recent study, López et al. analyzed 10 commercial exome enrichment kits from seven manufacturers. Designs of the targets covered 89% to 100% of CCDS regions. However, empirical evaluation of efficiency revealed that at 10x coverage depth, this fraction decreased to 76–95% and further to 47–92% at 20x [45]. To improve consistency across capture kits, we restrict SNV calling and joint genotyping to a common set of canonical protein-coding regions. This eliminates alternative contigs or kit-specific extensions of target exome regions, enforcing a shared observation space.

Although the exome capture kit is a major driver of forming the UMAP communities, we observed that samples assigned to SureSelectXTClin were located as two distinct groups corresponding to their dataset of origin (PRJNA824495 and EGAD00001002747). Our further investigation revealed potential annotation inconsistencies, suggesting a different kit was used for both of them. The first version of SureSelectXTClin (2014) was based on the SureSelectV5 panel, whereas SureSelectXTClinV2 (2017) was based on the SureSelectV6, according to the manufacturer’s specification [46]. In the UMAP space, samples from EGAD00001002747 (deposited in 2016) overlapped with V5 samples, while PRJNA824495 (deposited in 2022) aligned with V6 samples. This strongly suggests that the two datasets correspond to different versions of the SureSelectXTClin, fully explaining the spurious capture kit subdivision. These findings highlight the risk of relying solely on derived metadata labels and emphasize the importance of data-supported clustering to characterize the technical structure more precisely. The additional benefit of clustering is that we actually find the technically homogeneous groups sharing similar CWAF profiles, so if other major technical factors bias the data, they will also be captured.

Existing approaches address only selected components of the technical variability considered in this work. Standard WES pipelines, such as GATK, DRAGEN, or Sentieon, primarily differ in read and SNV processing steps. Thus, they can reduce the variability driven by tool-related factors, but they do not address gene-level under-detection biases caused by exome enrichment inefficiency. Similarly, coverage normalization techniques like downsampling or GC bias correction can reduce the variability in sequencing depth, but cannot recover the genomic regions that are systematically insufficiently enriched or absent from a particular capture kit design. The DeepVariant–GLnexus workflow was selected as an alternative to traditional pipelines based on previously reported benchmarking performance and its scalability in large cohorts [47,48,49]. In the context of multi-source WES data, joint genotyping provides a consistent representation of SNV sites across the cohort, reducing sample-specific calling variability, which is particularly important in terms of mitigating technical biases. However, the proposed batch effect correction strategy is not exclusively specific to DeepVariant and GLnexus tools. Starting from the cohort joint-genotyped VCF file, the workflow could be applied downstream of alternative WES processing pipelines. The current pipeline is dedicated to germline analysis, but it could also be upgraded to somatic analysis.

Despite the wide range of batch correction methods developed for transcriptomics, their application to SNV-derived features, such as CWAF, is limited. After normalization, gene expression levels can be treated as continuous variables, with batch effects often manifesting as mean and variance shifts, which enables their correction with statistical methods [50]. In contrast, SNV call data exhibit a more complex structure, containing various data types such as coordinates, genotypes, alleles, frequencies, or likelihoods. There are also numerous classes of genomic variation, including SNVs, indels, multi-nucleotide variants (MNVs), structural variants, and complex rearrangements [51]. Moreover, a single genomic variation can be represented in multiple ways [52]. After the variant aggregation, CWAF values follow a sparse, zero-inflated, multimodal distribution with peaks around expected allele ratios (0, 0.5, and 1). These characteristics violate the assumptions of commonly used methods. For example, ComBat requires approximately continuous, normally distributed data, with a ComBat-seq modification assuming a negative binomial distribution. The limma method assumes batch effects to be linearly additive. Furthermore, none of these methods explicitly account for the observed non-random missingness (MNAR), where the absence of a SNV often reflects a lack of detection.

Given these limitations, alternative approaches that account for the structure of SNV-derived data are required, particularly in terms of batch effects driven by the use of different exome enrichment kits. In this context, the proposed workflow addresses these biases by involving the enforcement of shared observation space, joint genotyping, genotype imputation, and data-driven clustering combined with MNAR-aware gene-level imputation. The results indicate that this strategy reduces gene detection discrepancies, thereby improving sample comparability in multi-source gene-level SNV data. Although UMAP embeddings are not intended as definitive evidence, they provide a useful representation of global and local data structure. No substantial sample mixing was observed; however, clusters exhibited reduced compactness and increased dispersion, suggesting a decreased differentiating signal. These findings are consistent with batch effect metrics, which indicate improved batch integration within the local neighborhood structure.

The genotype imputation accuracy is strongly determined by the quality and size of the reference panel. Although the 1kGP panel included in the pipeline is a widely used resource, derived from high-quality sequencing data, the sample size (n = 632) is limited. Larger reference panels, such as TOPMed, which include over 130,000 individuals, could provide substantially improved performance [53]. However, their integration into the proposed workflow is currently limited by the lack of readily accessible GRCh38-compatible releases to run locally. Also, genotype imputation does not replace direct experimental observation and imputed calls can carry uncertainty. We are using Beagle, which is state-of-the-art software for imputing sequencing data and was originally validated using 485,301 UK Biobank samples and 38,387 TOPMed samples, so we rely on its robustness and performance. The reliability of Beagle has been additionally demonstrated in independent benchmarking studies. Similar to our design, using Beagle with the 1000 Genomes Project reference panel, Homburger et al. reported imputation accuracy *r*^2^ > 0.9 at only 0.5× sequencing coverage [54]. Moreover, in our analyzed dataset, we recovered only 2% of missing genotypes, with imputation restricted to individual missing sample genotypes at loci already observed in the cohort. After aggregating to gene-level features, they are a minority and should not negatively impact the obtained scores.

In the proposed gene-level imputation method, Gaussian mixture modeling (GMM) was performed on the detection rate (DR). The rationale for using the DR is as follows: if for a given gene there are no SNVs in a given cluster, but there is at least one cluster with SNVs found for all samples, this gene is not efficiently captured by the analyzed cluster kit. Thus, the DR estimates the inefficiency of a specific kit for a given gene. Due to the sequencing errors and natural variation between patients with the same disease, we decided to relax the assumption and choose threshold values for low and high DRs. For this, it was planned to use a simple, distribution-based method, like z-score thresholding, but since the distribution of the DR is multimodal (as shown in Figure 4), GMM is used.

### 4.1. Limitations

Several limitations of this study should be acknowledged. First, the proposed workflow is strongly dependent on the variability of the analyzed cohorts. As the number and diversity of exome capture kits increase, the observable feature space expands, and more MNAR events can be properly identified. Furthermore, as the results of the kNN application show, the imputed values are inherently dependent on the composition of the dataset and can vary when the cohort structure changes, unlike reference-based methods. However, this also enables adaptation to the underlying data structure, which is beneficial for heterogeneous datasets. Regardless of the very small number of imputed CWAF values, there is a potential risk of over-imputation that may cause biological signal inflation. Next, even though 11 different datasets were analyzed, all of them consist of breast cancer patients of European ancestry. It is unknown how the data variability in different disease settings or ancestry will influence the results. To prove pipeline generalization capabilities, it should be validated using external benchmark datasets. However, there is no publicly available ready-to-go benchmark with similar data size and heterogeneity to the one analyzed here. Another limitation of the proposed approach is the clustering sensitivity to parameter selection. Overly coarse clusters may mask meaningful signals, while too granular clusters may lead to overcorrection. In addition, our workflow is primarily applicable to single-ancestry cohorts. A uniform population structure was necessary to enable a proper assessment of underlying technical variation, as CWAF is a direct transformation of AF, known to differ significantly across ancestral groups [55]. Inclusion of multiple ancestries would introduce a dominant genetic signal, as previously demonstrated [12]. So, in multi-ancestry settings, the MNAR masking algorithm, which is essentially based on comparing AF-derived features, may be confounded by differences between the genetic populations rather than true detection biases.

### 4.2. Future Directions

To address these limitations, future work should focus on incorporating the ancestry covariate into the framework. During the gene-level imputation, this would require restricting the MNAR masking and kNN imputation to samples with a shared ancestral background. This, in turn, would also facilitate the inclusion of larger and more diverse datasets, thereby improving the robustness and generalizability of the proposed workflow.

## Figures and Tables

**Table 1:** Analyzed datasets with the corresponding exome capture kits and aliases. SureSelect kits are manufactured by Agilent, whereas SeqCap kits are produced by Nimblegen (now Roche).

Dataset	Samples	Exome Capture kit	Kit Alias
PRJNA412025	10	SureSelect Human All Exon V4	SureSelectV4
BEAUTY	106		
PRJNA516884	9	SureSelect Human All Exon V5	SureSelectV5
EGAD00001002747	207 ^1^		
		SureSelect XT Clinical Research	SureSelectXTClin
PRJNA824495	38		
PRJNA1085200	19	SureSelect Human All Exon V6	SureSelectV6
PRJNA851929	8		
EGAD50000000770	49	SureSelect XT Human All Exon V7	SureSelectXTV7
TCGA	566 ^2^	SeqCap EZ Exome V2	SeqCapV2
		SeqCap EZ Exome V3	SeqCapV3
EGAD00001003137	4		
Yale	61	IDT xGen Exome Research Panel	IDT

^1^ SureSelectV5 = 90, SureSelectXTClin = 117; ^2^ SeqCapV2 = 457, SeqCapV3 = 109.

**Table 2:** Cluster assignments for genotype-imputed data, with regard to exome enrichment kits used. Numbers in the header reflect cluster numbers.

Capture Kit	0	1	2	3	4	5
IDT						61
SeqCapV2	261	1	195			
SeqCapV3	111		2			
SureSelectV4	1			114		1
SureSelectV5		90		4	3	2
SureSelectV6					26	1
SureSelectXTV7					49	
SureSelectXTClin	1	116			37	1
Total	374	207	197	118	115	66

## Data Availability

The datasets analyzed in this study were obtained from multiple sources, including publicly available repositories and controlled-access databases. Publicly available data were retrieved from the Sequence Read Archive (SRA) under accession numbers PRJNA412025, PRJNA516884, PRJNA824495, PRJNA1085200, and PRJNA851929. Controlled-access data were obtained from the European Genome-phenome Archive (accessions: EGAD00001002747, EGAD50000000770, EGAD00001003137), The Cancer Genome Atlas, and the Database of Genotypes and Phenotypes (BEAUTY, accession: phs001050.v1.p1). Access to controlled datasets requires authorization from the respective data access committees. Additional data from the Yale cohort are available on request from the corresponding author due to specific data use agreements. Several samples from the Yale cohort are deposited on the SRA database under accession number PRJNA1087680.

## Supplementary Materials

The following supporting information can be downloaded at https://www.mdpi.com/article/10.3390/mps9050125/s1. Figure S1. PARC clustering parameters analysis. Nine combinations of knn (20, 25, 30) and resolution (0.5, 1.0, 1.4) were compared on the same UMAP embedding. The final setting (knn = 30, resolution = 1.0) was selected as the smallest number of clusters preserving the major groups. Other settings with the same number of clusters differed only in the assignment of individual samples, indicating stability across major technical groups. Figure S2. Preservation of age-associated biological signal following gene-level imputation. (A) Comparison of limma T-statistics models testing age group effects (<50 vs. ≥50) while adjusting for technical cluster, before and after gene-level imputation. Pearson correlation quantifies concordance of age-associated effects. (B) Relationship between the absolute change in the T-statistic (|ΔT|) and the difference in gene detection rate across technical clusters. Table S1. BAM quality metrics for datasets provided as BAM files. Table S2. Sensitivity analysis of MNAR detection-rate thresholds. Fraction of imputed entries, LISI, and kBET values are shown for all nine combinations of low-detection threshold a and high-detection threshold b. Table S3. Top 10% most significant genes differentiating the clusters.

## Abbreviations

The following abbreviations are used in this manuscript:

1kGP	The 1000 Genomes Project
AF	Allele fraction
CCDS	Consensus coding sequence
CWAF	CADD-weighted allele fraction
DR	Gene detection rate
FDR	False discovery rate
FFPE	Formalin-fixed paraffin-embedded
kBET	k-nearest neighbor Batch Effect Test
kNN	k-nearest neighbors
LISI	Local inverse Simpson’s index
MNAR	Missing not at random
PARC	Phenotyping by accelerated refined community-partitioning
SNV	Single nucleotide variation
TCGA	The Cancer Genome Atlas
UMAP	Uniform manifold approximation and projection
WES	Whole-exome sequencing
WGS	Whole-genome sequencing

## Appendix A

**Table A1:** Annotation databases used in ANNOVAR.

Database	Version
RefGene	hg38 2020-08-17
ExAC	v0.3
gnomAD exomes	v4.1
1000 Genomes	2015-08
dbSNP	build 150
ClinVar	2024-12-1
dbNSFP	v4.7a
