# Supplementary material for: A Practical Workflow for Correcting Kit-Specific Effects in Whole-Exome Sequencing Data
Source: Methods Protoc. 2026 Aug 25;9(5):125. doi: 10.3390/mps9050125 (PMC13600025; doi:10.3390/mps9050125)
Supplement: Supplementary file 1 [file mps-09-00125-s001.zip › Supplementary figures.pdf]

Supplementary figures

Supplementary Figure S1

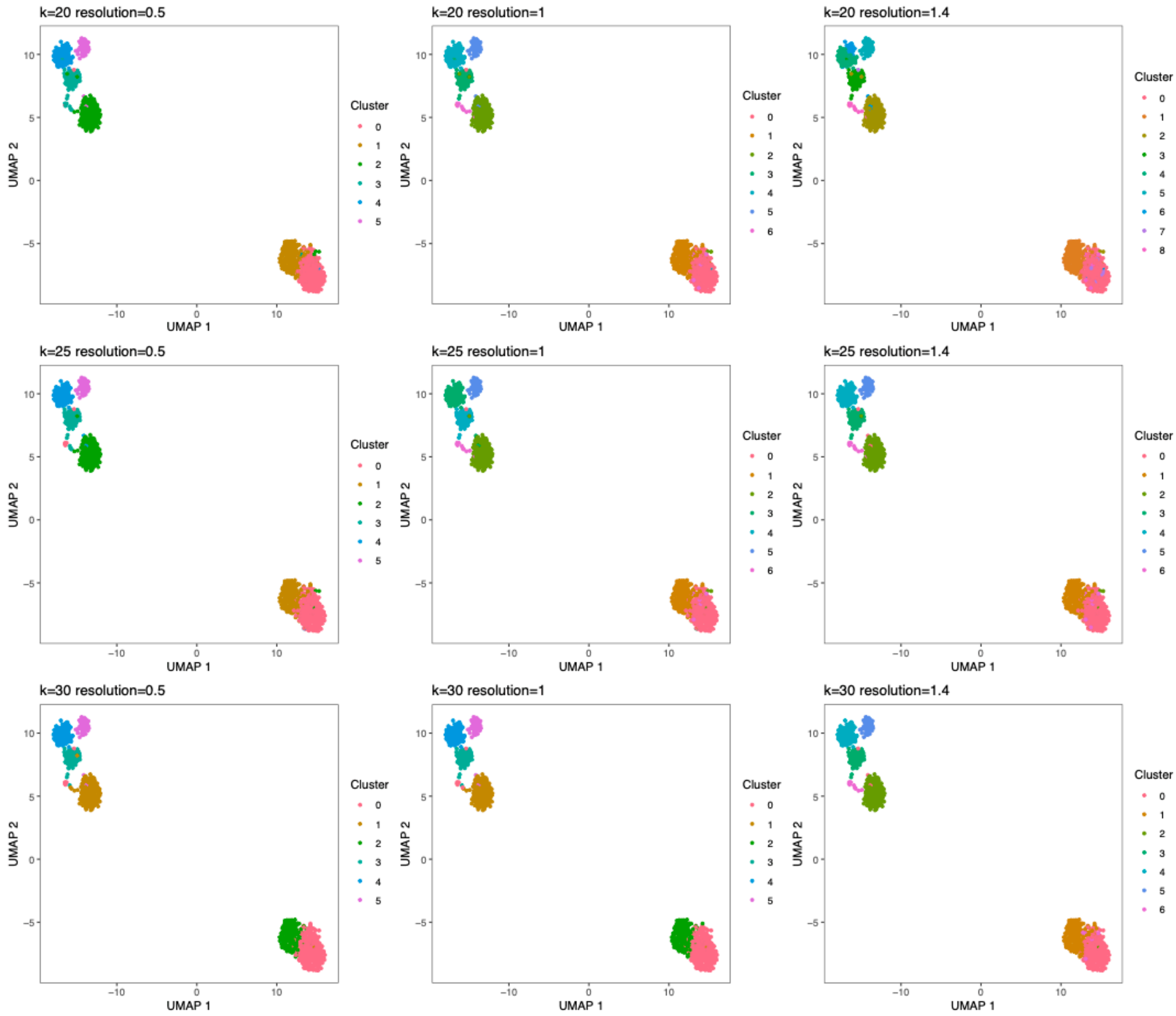

Figure S1 PARC clustering parameters analysis. Nine combinations of *knn (20, 25, 30)* and *resolution (0.5, 1.0, 1.4)* were compared on the same UMAP embedding. The final setting *(knn = 30, resolution = 1.0)* was selected as the smallest number of clusters preserving the major groups. Other settings with the same number of clusters differed only in the assignment of individual samples indicating stability across major technical groups.

Supplementary Figure S2

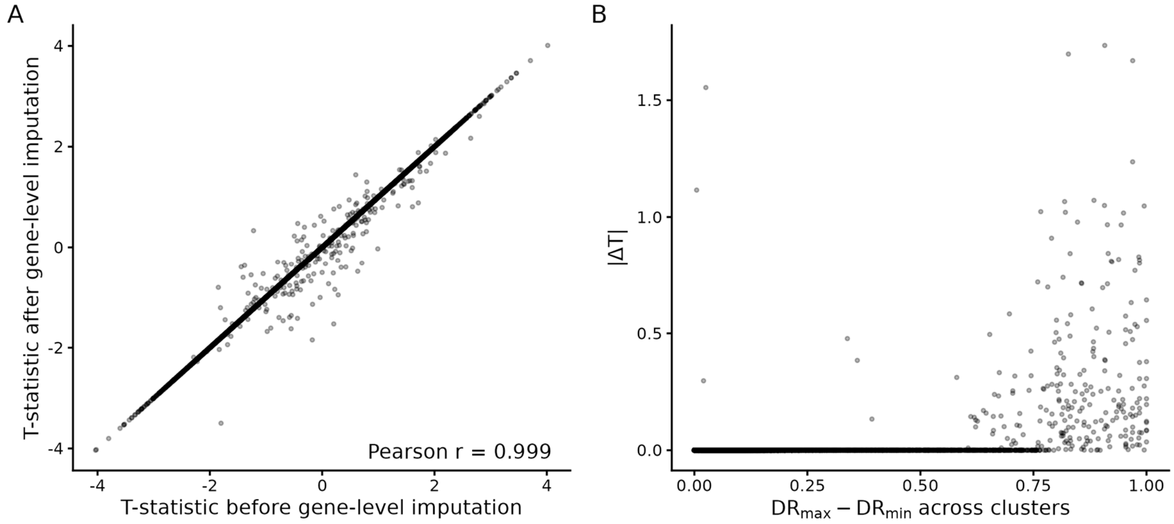

Figure S2. Preservation of age-associated biological signal following gene-level imputation. (A) Comparison of limma T-statistics models testing age group effects (<50 vs ≥50) while adjusting for technical cluster, before and after gene-level imputation. Pearson correlation quantifies concordance of age-associated effects. (B) Relationship between the absolute change in the T-statistic (|ΔT|) and the difference in gene detection rate across technical clusters.
